# Diversification of the expanded teleost-specific toll-like receptor family in Atlantic cod, *Gadus morhua*

**DOI:** 10.1186/1471-2148-12-256

**Published:** 2012-12-29

**Authors:** Arvind YM Sundaram, Viswanath Kiron, Joaquín Dopazo, Jorge MO Fernandes

**Affiliations:** 1 Faculty of Biosciences and Aquaculture, University of Nordland, Bodø 8049, Norway; 2 Bioinformatics Department, Centro de Investigación Príncipe Felipe, Valencia, 46012, Spain

**Keywords:** Atlantic cod, Toll-like receptors, TLR, Innate immunity, Positive selection, Thermal stress, Neofunctionalisation

## Abstract

**Background:**

Toll-like receptors (Tlrs) are major molecular pattern recognition receptors of the innate immune system. Atlantic cod (*Gadus morhua*) is the first vertebrate known to have lost most of the mammalian Tlr orthologues, particularly all bacterial recognising and other cell surface Tlrs. On the other hand, its genome encodes a unique repertoire of teleost-specific Tlrs. The aim of this study was to investigate if these duplicate Tlrs have been retained through adaptive evolution to compensate for the lack of other cell surface Tlrs in the cod genome.

**Results:**

In this study, one *tlr21*, 12 *tlr22* and two *tlr23* genes representing the teleost-specific Tlr family have been cloned and characterised in cod. Phylogenetic analysis grouped all *tlr22* genes under a single clade, indicating that the multiple cod paralogues have arisen through lineage-specific duplications. All *tlr*s examined were transcribed in immune-related tissues as well as in stomach, gut and gonads of adult cod and were differentially expressed during early development. These *tlr*s were also differentially regulated following immune challenge by immersion with *Vibrio anguillarum*, indicating their role in the immune response. An increase in water temperature from 4 to 12°C was associated with a 5.5-fold down-regulation of *tlr22d* transcript levels in spleen. Maximum likelihood analysis with different evolution models revealed that *tlr22* genes are under positive selection. A total of 24 codons were found to be positively selected, of which 19 are in the ligand binding region of ectodomain.

**Conclusion:**

Positive selection pressure coupled with experimental evidence of differential expression strongly support the hypothesis that teleost-specific *tlr* paralogues in cod are undergoing neofunctionalisation and can recognise bacterial pathogen-associated molecular patterns to compensate for the lack of other cell surface Tlrs.

## Background

Toll-like receptors (TLRs) are an integral part of the innate immune system in all organisms and form one of the first lines of defence against invading pathogens. They are a class of pathogen recognition receptors (PRRs) that elicit specific responses against pathogens upon recognising pathogen-associated molecular patterns (PAMPs) [1]. Most TLRs are type-I transmembrane proteins that are composed of three domains: an intracellular Toll/interleukin-1 receptor (TIR) domain, a transmembrane region and an extracellular domain. The TIR domain is highly conserved across all transmembrane TLRs and initiates signal transduction, while the variable extracellular domain is composed of leucine-rich repeats (LRR) motifs that are involved in recognising specific PAMPs [2]. To date, 21 different TLRs have been identified across numerous vertebrates [3]. Based on phylogenetic analyses, they are organised in six major families: TLR1 (TLRs 1, 2, 6, 10 and 14), TLR3, TLR4, TLR5, TLR7 (TLRs 7, 8, 9) and TLR11 (TLRs 11 to 13 and TLRs 21 to 23) [3]. Avian, amphibian and teleost genomes encode for most of the mammalian orthologues, as well as additional TLRs [4-6]. Tlr15 has been identified only in birds, whereas Tlr18, Tlr19 and Tlr20 are found in teleosts. Tlr21, Tlr22 and Tlr23 are generally termed as ‘teleost-specific Tlrs’, since they are present in several teleost taxa [7]. Nevertheless, a putative Tlr21 has been identified in chicken (*Gallus gallus*) [8], while Tlr21 and Tlr22 have been found in *Xenopus tropicalis*[5].

Even though the role of TLRs in detecting pathogens is well documented, these molecules are also known to be activated by endogenous agonists and to be involved in other biological functions. Early studies in *Drosophila melanogaster* have demonstrated that they control the formation of the dorso-ventral axis during embryogenesis [9]. Heat shock proteins, inflammatory mediators and fragments of molecules from extracellular matrix, which are mainly generated in response to stress or as a consequence of tissue injury, have the potential to activate TLRs [2].

In spite of a large degree of conservation between teleost TLRs and their mammalian orthologues, there are some differences in signalling and their ability to recognise PAMPs [4]. Unlike in mammals, there is not always a one to one relationship between teleost Tlr families and the PAMPs that they recognise. Immunostimulation experiments have revealed that several teleost TLRs respond to PAMPs from bacterial and viral origin [4]. In particular, the teleost-specific Tlr22 is known to recognise dsRNA in tiger pufferfish, *Takifugu rubripes*[10], but it also responds to other PAMPs from Gram-positive and Gram-negative bacteria in other teleosts [4,11-13].

The recently published Atlantic cod (*Gadus morhua*) genome draft has uncovered a unique feature of its immune system: the absence of the genes encoding for major histocompatibility complex (MHC) II, CD4 and invariant chain, which are key components of the adaptive immune system in jawed vertebrates [14]. However, this fish has a large number of MHC I genes and a unique repertoire of TLR families in its genome. The cod genome encodes four of the mammalian homologues (*tlr3*, *tlr7*, *tlr8* and *tlr9*), and all three teleost-specific *tlr*s (*tlr21, tlr22* and *tlr23*), representing three of the six TLR families. It has lost all cell surface receptors as well as bacterial recognising mammalian homologues from the TLR1, TLR4 and TLR5 families. A single copy of *tlr14* has been identified in the cod genome, but the ligand specificity of this Tlr family member is still unknown.

Gene duplication is a major force of adaptive genome evolution, since it allows duplicate genes to explore different aspects of the multidimensional functional space [15]. Even if most duplicates degenerate into pseudogenes (nonfunctionalisation or pseudogenisation) within 50 million years following the duplication event, a remarkable number of gene duplicates are found in vertebrate genomes [16]. One of the main mechanisms that account for the increased probability of retaining duplicate genes is the acquisition of a novel function (neofunctionalisation) by one of the copies, which is no longer required to maintain the original functions [17]. An alternative model, which is not incompatible with subsequent neofunctionalisation, is the sharing of ancestral functions between gene duplicates (subfunctionalisation), namely partitioning of spatio-temporal expression domains [18]. The relative contribution of neofunctionalisation and subfunctionalisation in early vertebrate evolution is still a matter of controversial debate and little is known about the role of adaptive and/ or non-adaptive pressures in the maintenance of duplicate genes (reviewed in [19]). One of the factors that make it difficult to distinguish these processes is the long divergence time, which clouds direct tests of selection on ancient evolutionary events. Analyses of more recent duplications, such as the ones found in some teleost lineages, may prove useful to overcome this issue [20].

The expanded teleost-specific Tlr family in cod is so far unique amongst teleosts and provides a good model to better understand how and why so many duplicate genes have been retained during vertebrate evolution. It is plausible that these multiple teleost-specific paralogues are retained through adaptive evolution to compensate for the lack of other cell surface Tlrs in the cod genome. To address this hypothesis, we have examined the molecular evolution and differential expression of all teleost-specific Tlrs present in the current cod genome assembly.

## Methods

### Sources of biological samples

#### Tissue and embryo samples from naïve fish

Two-year old Atlantic cod (Codfarmers ASA, Norway), reared in land based tanks at Mørkvedbukta research station (University of Nordland, Norway) were used for this study. The flow-through rearing system was supplied with sea water at 7–8°C and the fish were fed daily with a commercial diet (Amber Neptun, Skretting AS, Stavanger, Norway). Adult fish were humanely killed by immersion in an anaesthetic bath containing 0.5 g·L^-1^ tricaine methanesulfonate (Sigma) in accordance with the national guidelines detailed in the “Norwegian Regulation on Animal Experimentation” (Forsøksdyrutvalget, Norway). Head-kidney, kidney, spleen, liver, stomach, gut, heart, gills, muscle, skin, brain, blood and gonads were collected, snap-frozen in liquid nitrogen and stored at −80°C for subsequent RNA extraction.

Cod eggs for this study were kindly provided by Codfarmers ASA (Norway). Unfertilised eggs were immediately frozen in liquid nitrogen and stored at −80°C until RNA extraction. Eggs from individual cod spawning pairs were artificially fertilised in drum-filtered (30 μm) UV treated seawater (7°C) and maintained without aeration at a density of 10 mL·L^-1^. Up to one third of the seawater was replaced on a daily basis, so as to keep the oxygen concentration above 6.5 mg·L^-1^. Embryos at different developmental stages (4-cells, 16-cells, oblong, 25% epiboly, 75% epiboly, 10-somite, 30-somite and golden eye) and larvae (hatched, bladder stage, hindgut stage and first feeding) were observed under an optical microscope and approximately 50 specimens from each stage were collected, snap-frozen in liquid nitrogen and stored at −80°C for further analysis.

### Tissue samples from fish exposed to a bacterial pathogen

This experiment was conducted at the Institute of Marine Research, Norway. One hundred and twenty adult fish with an average weight of 60 g were equally distributed in three 250 L tanks, which were part of a flow-through system that was supplied with sea water at 7–8°C. The fish were maintained in this system for a period of five weeks prior to the challenge experiment. They were fed daily with a fishmeal based feed [21] at 1.5% (w/w) of their body weight every day. Prior to bacterial challenge, initial control samples were collected from six fish, two per tank. Thereafter, the water flow was stopped and fish in all three tanks were subjected to bath challenge with *V. anguillarum* strain H610 at a concentration of 2.6·10^7^ cfu·ml^-1^ for 1 h [21]. Post-challenge samples were collected at 4 (4 hpc) and 48 (48 hpc) h after exposure. The samples collected included head-kidney, gills and spleen, which were immediately snap-frozen in liquid nitrogen and maintained at −80°C for further analysis.

### Temperature stress

The temperature stress experiment was conducted at the indoor facilities of Mørkvedbukta research station. Fifty adult cod with a mean weight of 263 ± 50 g were evenly distributed in two 500 L tanks and fed daily (Amber Neptun, Skretting AS, Norway) to 1.5% (w/w) of their body weight. Seawater at 4°C was supplied to the rearing tanks and the fish were allowed to acclimatise for a period of one week prior to the temperature stress experiment. Initial control samples were collected at the start of the experiment. Water temperature was then increased from 4°C to 12°C at a rate of 2°C·h^-1^ and the first post-stress samples were collected at 4 h (4 hps) when the water temperature reached 12°C. Fish were further maintained at 12°C and the final sample was collected after 72 h (72 hps). Three fish were taken from each tank at each sampling point (n=6) and humanely killed as above. Head-kidney and spleen were immediately dissected, snap-frozen in liquid nitrogen and stored at −80°C prior to RNA extraction.

### RNA extraction and cDNA synthesis

The above samples were lysed in Lysing Matrix D (MP Biomedicals, USA) and total RNA extracted using QIAzol (Qiagen, Netherlands) according to the manufacturer’s instructions. Quality and quantity of total RNA were assessed by agarose electrophoresis and spectrophotometry (NanoDrop, Thermo Scientific, USA), respectively. Complementary DNA was synthesised using the Quantitect reverse transcriptase kit (Qiagen, Netherlands). Total RNA was treated with gDNA wipeout buffer provided in the reverse transcriptase kit to remove any traces of genomic DNA. Luciferase mRNA (Promega, USA) was used as an external control, as previously reported [22].

### Cloning of Atlantic cod tlr21, tlr22 and tlr23 genes

Tlr21, Tlr22 and Tlr23 protein sequences from zebrafish (*Danio rerio*), stickleback (*Gasterosteus aculeatus*), green-spotted pufferfish (*Tetraodon nigroviridis*) and tiger pufferfish were used as queries in Ensembl BLAST searches (http://www.ensembl.org) against the cod genome (gadMor1 v67.1). In order to predict gene sequences, contigs and scaffolds, the above BLAST hits were further analysed using the AUGUSTUS gene prediction server at University of Greifswald [23]. Based on predicted coding sequences, primers were designed to amplify partial coding regions of the respective paralogues (Additional file 1). Total RNA from head-kidney, kidney, spleen and gills were pooled, reverse transcribed as above and used as PCR template. Following amplification by PCR, the products of interest were analysed using gel electrophoresis, purified, cloned and sequenced as described elsewhere [24]. The GeneRacer kit with SuperScript III RT (Invitrogen, USA) was used to perform RACE PCR in order to obtain full length cDNA sequences. Outer and inner gene specific primers for both 5^′^ and 3^′^ RACE were designed based on the partial sequences obtained above. RACE cDNA was synthesised as per the manufacturer’s protocol using total RNA pooled from head-kidney, kidney, spleen and gills. PCR products were cloned and sequenced using the primers listed in Additional file 1.

### Sequence analysis

All sequences were analysed and assembled in CodonCode Aligner v3.7.1 (http://www.codoncode.com/aligner) using default settings and their identity determined by BLASTN similarity searches against the NCBI non-redundant database. Nucleotide sequences were analysed for a Kozak consensus sequence to identify the start codon using ATGpr (atgpr.dbcls.jp) and the corresponding protein sequences were obtained using Translate (web.expasy.org/translate). Nucleotide data were submitted to Genbank under the accession numbers shown on Table 1. Cod *tlr* sequences and their teleost homologues (Additional file 2), as well as their corresponding protein sequences, were aligned with MatGat 2.02 (http://www.bitincka.com/ledion/matgat) using BLOSUM50 to generate identity and similarity matrices. Protein domains were predicted by ScanProsite (prosite.expasy.org/scanprosite) and LRR motifs were mapped manually on the protein sequence based on the corresponding tiger pufferfish Tlrs [25]. Intron-exon boundaries were identified using the Ensembl cod genome sequence and Spidey (http://www.ncbi.nlm.nih.gov/spidey). Synteny analysis was performed manually based on the Ensembl assemblies of stickleback (v67.1), tiger pufferfish (v67.4), green-spotted pufferfish (v67.8), zebrafish (v67.9) and medaka, *Oryzias latipes* (v67.1).

**Table 1:** **Teleost-specific *tlr*s of Atlantic cod**

**Gene name**	**Accession number**	**Chromosomal Location**	**Sequence length (bp)**	**5**^**′**^**-UTR**	**CDS**	**3**^**′**^**-UTR**	**Protein length (aa)**
*tlr21*	JX074771	GeneScaffold_1988 contig373731	3047	134	**2913**	**-**	**970**
*tlr22a*	JX074772	GeneScaffold_1177 contig165664	1654	-	1654	-	551
*tlr22b*	JX074773	GeneScaffold_1177 contig165665	**3406**	**262**	**2829**	**315**	**942**
*tlr22c*	JX074774	GeneScaffold_1176 contig885687	2408	-	2408	-	802
*tlr22d*	JX074775	GeneScaffold_1176 contig165725	**3252**	**229**	**2880**	**143**	**959**
*tlr22e*	JX074776	GeneScaffold_1177 contig885683	1612	**252**	1360	**-**	453
*tlr22f*	JX074777	scaffold03378 contig96110	2707	**232**	2475	**-**	825
*tlr22g*	JX074778	GeneScaffold_1685 contig343097	**3082**	**272**	**2529**	**281**	**842**
*tlr22h*	JX074779	scaffold00128 contig05698	2847	**250**	2597	**-**	865
*tlr22i*	JX074780	contig536615	**3219**	**250**	**2865**	**104**	**954**
*tlr22j*	JX074781	contig520640	2149	-	2149	-	716
*tlr22k*	JX074782	GeneScaffold_351 contig605495	384	**-**	293	**91**	96
*tlr22l*	JX074783	GeneScaffold_351 contig892392	2706	**-**	2523	**183**	840
*tlr23a*	JX074784	scaffold12300 contig717163	**3427**	**340**	**2850**	**237**	**949**
*tlr23b*	JX074785	contig12242	**2165**	**131**	**1737**	**297**	**578**

Full length sequences are represented in bold.

### Phylogenetic inference

A total of 41 sequences from 14 teleosts (Additional file 2) were used to perform the phylogenetic analysis to elucidate the evolution of teleost *tlr*s. MUSCLE (http://www.ebi.ac.uk/Tools/msa/muscle) was used to align cDNA sequences and the best nucleotide substitution model was identified using MrModelTest v2.3 [26] and PAUP* v4.0b10 [27], as reported [28]. The best model to describe the data was identified based on the Akaike information criterion (AIC). Maximum likelihood phylogenetic analysis was carried out with PhyML [29] and Bayesian inference was performed as detailed elsewhere [28]. The multiple sequence alignment used for phylogenetic reconstruction and corresponding tree have been submitted to TreeBASE (http://www.treebase.org/) under the accession ID 13554.

### Quantification of gene expression

#### Primer design

Specific primers were designed to quantify the expression of Atlantic cod *tlr21*, *tlr22* and *tlr23* paralogues using qualitative RT-PCR as well as real-time PCR (qPCR) (Table 2). In RT-PCR, *eef1a* was used as an internal reference gene for tissue distribution analysis while *luciferase* was used as an external control to determine expression across developmental stages, as it has been shown that expression of commonly used housekeeping genes is not stable during this period, especially if it encompasses the maternal-zygotic transition [22]. *Eef1a* and *ubi* were used as reference genes for qPCR. Whenever possible, primers were designed across intron-exon boundaries and screened for hairpins, homo- and cross-dimers using Netprimer (http://www.premierbiosoft.com/netprimer).

**Table 2:** **Primers used for semi quantitative (RT-PCR) and real-time PCR (qPCR) of teleost-specific *tlr*s in Atlantic cod**

**Gene Name**	**qPCR primer (Forward and Reverse) (5**^**′**^**-3**^**′**^**)**	**Amplicon (bp)**	**RT-PCR/qPCR annealing (°C)**	**Efficiency (%)**
*tlr21*	CGTTACAATCGCATCCTCTCAG GCTGCTCCACAACTCAGTCAAG	177	58/60	110
*tlr22a*	GCAGGAAGTTCTGGAGACATTTA TCATTCACATTGGAGCACAAGTG	186	58/60	98
*tlr22b*	GAGTTGGACTTTGGGACGAA ACATTCCTGACGGCACAAG	128	58/60	125
*tlr22c*	TCAGTTCCCAATGCCGTAAG ACACAGTCCTTTAGAACCAAGACAC	155	58/62	130
*tlr22d*	AGAGGAGGGTATGTTTGATGGC TGTTCGCTAAGTTCCGCAGTT	152	58/62	116
*tlr22e*	CCAACCTCACAAGATTGAACCT GCAAGCGACAACCACTGATA	120	58/60	115
*tlr22f*	CGCTTAGACCTGAGACACAACTT AATCCATCAAACATACCCTCCTC	131	58/64	91
*tlr22g*	GCAGCAAACGAGATGTCCAC TCTCCCAGACGATACCATTCTC	178	58/64	116
*tlr22h*	GCTTAGACCTGACACGCAACA AAGCCAGACGCAGTTCAATG	159	58/62	130
*tlr22i*	GCATCGGTAGAGCCTATTCTGA GAAATTGGTCCGCTTATGAGA	102	58/64	111
*tlr22j*	TGTGATTAGAGAACCAGTGATGCT TGTGTCTGCTTGTTTGTGATTACC	129	58/62	92
*tlr22k*	TCCTACAATGGCAACTGGTCTAC CCCAGCCCTCGTCGTTTG	129	58/60	88
*tlr22l*	CTCTTAGGCTGCTTAACACTTTAATC TGGATAGATAGATAACGCTGAGACG	171	58/60	104
*tlr23a*	CCTTCGGCTACCACTTCCTG GCCTCGCTCGTCCTCCA	188	58/62	110
*tlr23b*	GACTCCAATTTCCTCTGCTTCA GGTGCTGCTCATTATTCTTCCT	163	58/64	94
*luciferase*	TCATTCTTCGCCAAAAGCACTCTG AGCCCATATCCTTGTCGTATCCC	149	58/58	98
*eef1a*	CACTGAGGTGAAGTCCGTTG GGGGTCGTTCTTGCTGTCT	142	58/58	110
*ubi*	GGCCGCAAAGATGCAGAT CTGGGCTCGACCTCAAGAGT	69	69/60	92

### Qualitative RT-PCR (RT-PCR)

Gene expression across tissues and developmental stages for Atlantic cod *tlr21*, *tlr22* and *tlr23* was determined using RT-PCR. Recombinant Taq DNA polymerase (VWR, USA) was used for RT-PCR with the following thermocycling parameters: 95°C: 2 min, 35 cycles of (95°C: 15 sec, annealing temperature (Table 1): 30 sec and 72°C: 2 min) and 72°C: 7 min. Amplification was carried out in Bio-Rad C1000 thermocycler (Bio-Rad, USA). Samples were analysed by electrophoresis on 1.5% (w/v) gels and then visualised and photographed using the Kodak Gel Logic 200 Imaging System (Carestream, USA).

### Real-time PCR (qPCR)

Quantification of gene expression was performed by real-time PCR with SYBR green chemistry on a LightCycler 480 (Roche, USA), as detailed elsewhere [11]. A dissociation step with a gradient from 65°C to 97°C was performed to check the specificity of the qPCR reaction and the absence of primer dimers. Specificity was further confirmed by Sanger sequencing of qPCR products. C_T_ values were calculated with a fluorescence threshold of 0.5 and the average of two technical replicates was used to calculate relative gene expression. Data were normalised against *eef1a* and *ubi* expression using geometric normalisation factors obtained from GeNorm (http://medgen.ugent.be/genorm/), as previously described [30]. Relative gene expression against the initial control sample was determined and statistical analysis was performed by one-way ANOVA with Tukey’s HSD post-hoc tests using the SigmaPlot 12.0 (Systat Software Inc., USA). When the data did not meet normality or equal variance requirements, a Kruskal-Wallis one-way ANOVA by ranks and median tests was performed. Significance levels were set at *P* < 0.05. The sample size was too small to exclude a tank effect but there was no obvious pattern of differential gene expression in one particular tank.

### Tests of selection pressure and divergence

All complete and partial *tlr22* paralogues were used for selection pressure analysis, except *tlr22a*, *tlr22e* and *tlr22k*, since these genes had only partial sequences of 1654, 1360 and 293 bp, respectively. Coding sequences of the other nine *tlr22* paralogues were aligned with MUSCLE and a codon alignment was performed using the Codon Align software (http://www.hiv.lanl.gov). The N-terminal portion of the codon aligned sequences was too variable and hence 210 bp of this region were removed prior to positive selection tests. Similarly, the C-terminal region coding for TIR domain was not included in the analysis, as it is highly conserved across all known transmembrane TLRs. Instead, a codon alignment comprising 75% (2169 bp) of the total CDS and without stop codons was used. The best nucleotide substitution model was selected using MrModelTest v2.3 [26] and PAUP* v4.0b10 [27] based on AIC. Differences in sequence diversity between the regions that code for different domain structures were examined by calculating the average number of synonymous (dS) and non-synonymous (dN) substitutions, insertions and deletions in the codon alignments using SNAP [31].

Codon based Z-tests of selection were performed to test the hypothesis of positive selection in MEGA4 [32] using the modified Nei-Gojobori method (Jukes-Cantor) and calculating the variance with 1000 bootstrap replicates [33]. Evolutionary distances between the nine Tlr22 paralogues were estimated by Tajima’s relative rate test [34]. Each pair of paralogues was compared with Tlr22b as outgroup, since it was the most distant Tlr22 paralogue for which the complete sequence was available. In addition, tests for positive selection were performed using the maximum likelihood methods implemented in the CODEML program of PAML, as detailed elsewhere [35]. The dN/dS ratio (ω) was calculated using models M0 (neutral), M1 (nearly neutral), M2 (positive selection), M7 (beta) and M8 (beta & ω). Models were compared against each other using likelihood ratio tests (LRTs). Bayesian posterior probabilities (p) were calculated for positively selected sites using naive empirical Bayes (NEB) and Bayes empirical Bayes (BEB). REL, FEL and SLAC analyses were carried out in Datamonkey (http://www.datamonkey.org) to calculate ω values for each codon, along with the corresponding probability values [36].

## Results

### Expanded teleost-specific tlrs in Atlantic cod

**Figure 1:**
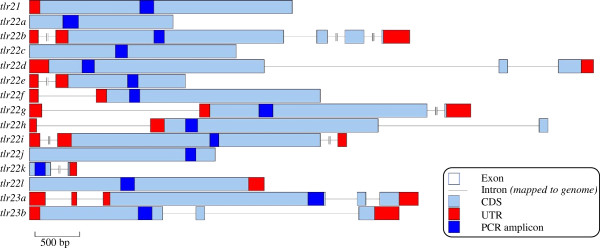
**Gene structure of teleost-specific *tlr*s in Atlantic cod.** Graphical representation of Atlantic cod *tlr21*, *tlr22* and *tlr23* gene structures. Exons and UTRs are represented in light blue and red, respectively. Introns are indicated by continuous lines. PCR amplicons are highlighted in dark blue. Scale bar represents 500 bp.

Homology searches for *tlr21*, *tlr22* and *tlr23* paralogues in the cod genome assembly identified 15 open reading frames that encode proteins with homology to these teleost-specific *tlr*s. *In silico* gene prediction analysis confirmed the presence of one *tlr21*, 12 *tlr22* paralogues and two *tlr23* paralogues, all encoding a typical Tlr protein (Table 1). A partial *tlr21* cDNA of 3047 bp was sequenced, including the 134 bp 5^′^-UTR and the 2913 bp complete coding region corresponding to a 970 aa protein. Cod Tlr21 shares more than 50% identity with its orthologues in zebrafish, tiger pufferfish and medaka, as well as with Tlr21a and Tlr21b of orange-spotted grouper (*Epinephelus coioides*). Based on the genome assembly, the *tlr21* partial sequence was found to be encoded by a single exon (Figure 1). Full length cDNA sequences along with the 5^′^- and 3^′^-UTR regions were obtained for four *tlr22* paralogues. *Tlr22b*, *tlr22d*, *tlr22g* and *tlr22i* were 3406, 3252, 3082 and 3219 bp long and encoding 942, 959, 842 and 954 aa proteins, respectively. They were composed of five, three, three and three exons, respectively (Figure 1). At the protein level, they are 62 to 75% identical to each other and share up to 73% similarity with other teleost Tlr22 proteins. Partial coding sequences for seven of the *tlr22* paralogues were obtained either with or without the UTR regions from a minimum length of 1612 bp up to 2847 bp, encoding partial proteins of 453 aa to 865 aa. In the case of *tlr22k*, it was only possible to obtain a short sequence of 384 bp, including the 3^′^-UTR and coding for a 96 aa partial protein (Table 1). Complete cDNA sequences were determined for both *tlr23* paralogues in cod. *Tlr23a* was 3427 bp while *tlr23b* was only 2165 bp. *Tlr23a* and *tlr23b* were encoded by 5 and 3 exons, respectively (Figure 1), corresponding to proteins of 949 and 578 aa, respectively. At the nucleotide level, *tlr23a* and *tlr23b* were 45% identical to each other and shared 47% identity at the protein level with tiger pufferfish and green-spotted pufferfish Tlr23.

**Figure 2:**
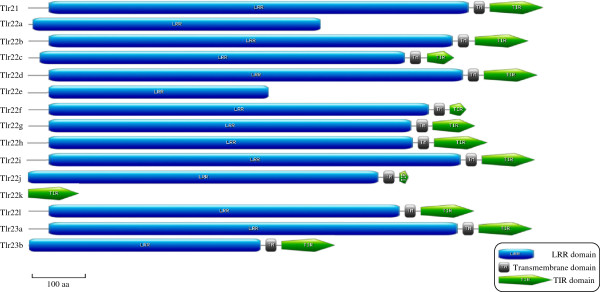
**Protein domain structure of teleost-specific Tlrs in Atlantic cod.** Graphical representation of Atlantic cod Tlr21, Tlr22 and Tlr23 protein structure predicted by ScanProsite. LRR ectodomain, transmembrane domain and TIR domain are represented by blue, grey and green colored shapes, respectively. Scale bar indicates 100 aa.

In general, all *tlr*s analysed in this study had an N-terminal LRR domain, a transmembrane domain and a C-terminal TIR signalling domain (Figure 2). Leucine rich repeats (LRRs) were mapped manually and the LRR C-terminal (LRRCT) domain was also identified. *Tlr21* contained 27 LRRs and a typical CxCx_24_Cx_15_C motif in its LRRCT domain. Full length cDNAs from *tlr22b*, *tlr22d*, *tlr22g* and *tlr22i* encoded for 27 LRRs and had a CxCx_24_Cx_18_C motif at its LRRCT domain. Tlr23a and Tlr23b had CxCx_24_Cx_18_C at their LRRCT domain with 27 and 14 LRRs, respectively.

### Synteny and phylogenetic analysis of teleost-specific tlrs in cod

**Figure 3:**
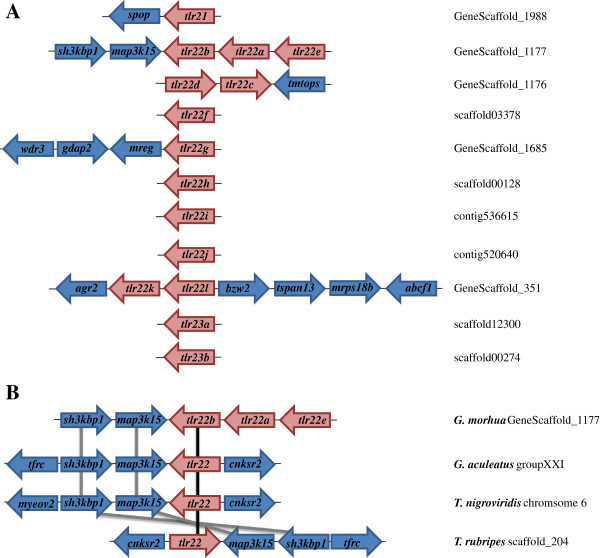
**Partial synteny map of the genomic region surrounding teleost-specific Atlantic cod *tlr *genes. A**. Partial map of the genomic regions surrounding the Atlantic cod *tlr21*, *tlr22* and *tlr23* paralogues. Their genomic location based on the current draft genomic sequence of Atlantic cod (gadMor1 v67.1) is also indicated. **B**. Partial synteny map between cod *tlr22a*, *tlr22b* and *tlr22e* and *tlr22* of stickleback (*G. aculeatus*), green-spotted pufferfish (*T. nigroviridis*) and tiger pufferfish (*T. rubripes*). *Tlr22* paralogues are connected by black lines while genes in their vicinity are connected by grey lines to show synteny amongst these four teleosts. Genes are not represented to scale.

Most cod *tlr*s were mapped to single contigs (Table 1). *Tlr22a*, *tlr22b* and *tlr22e* were present in the same chromosomal region (GeneScaffold_1177), which was syntenic in stickleback, tiger pufferfish and green-spotted pufferfish *tlr22* (Figure 3). *Tlr22c* and *tlr22d* were found in GeneScaffold_1176 and *tlr22k* and *tlr22l* were both in GeneScaffold_351 along with other genes, but there was no identifiable synteny in these regions across other teleost genomes (Figure 3).

**Figure 4:**
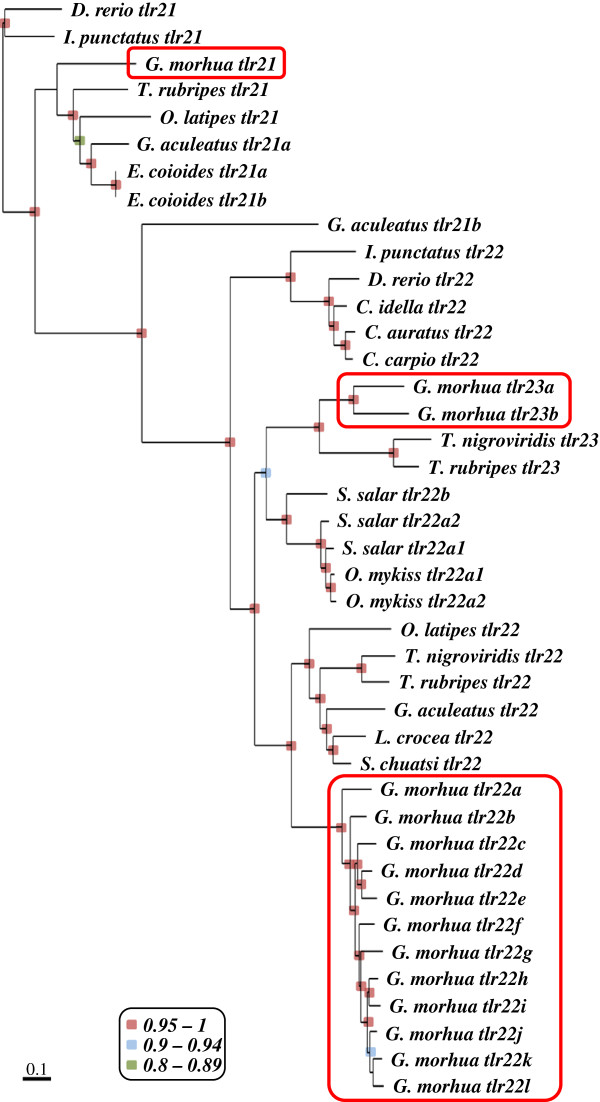
**Phylogeny of teleost-specific *tlr*s.** Unrooted phylogenetic tree of teleost-specific *tlr*s – *tlr21*, *tlr22* and *tlr23.* Numbers at the nodes indicate posterior probability values from Bayesian inference. Posterior probability values were calculated for each node by Bayesian analysis based on 250,000 generations. Samples were collected every 100 generation and a consensus tree was built after burning the initial 1,250 trees. Only probability values above 0.8 are indicated: 0.95 to 1 shaded in red, 0.9 to 0.94 in blue and 0.8 to 0.89 in green, respectively. Atlantic cod genes are highlighted within red boxes.

Bayesian inference from 41 tlr21, tlr22 and tlr23 sequences from 15 teleost species generated a consensus phylogenetic tree that was identical to the maximum likelihood one (Figure 4). All *tlr21* genes were grouped under a single clade, while *tlr22* and *tlr23* formed a separate cluster. Stickleback *tlr21a* clustered with other teleost *tlr21* genes, while *tlr21b* seemed to have arisen from a recent duplication and was more closely related to teleost *tlr22*. It is noteworthy that all *tlr22* from cod clustered under a single clade, while the two *tlr23* paralogues clustered along with their homologues from Tetraodontidae. As expected, the *tlr22* paralogues encoded by salmonids, such as Atlantic salmon and rainbow trout, were grouped together and corresponded to closely related paralogues, which have probably arisen from the salmonid tetraploidisation. Amongst the cod *tlr22* paralogues that are adjacent in the genome (Figure 3), only *tlr22k* and *tlr22l* clustered together, whereas *tlr22a*, *tlr22b* and *tlr22e* or *tlr22c* and *tlr22d* did not. *Tlr22* encoded by basal teleosts belonging to the Ostariophysi superorder clustered as a separate clade followed by Salmonidae and higher teleosts from the Acanthopterygii superorder. Unexpectedly, cod *tlr22* paralogues were more distant from the ancestral *tlr22* sequence than their Acanthopterygii orthologues.

### Expression profiles of teleost-specific tlrs in adult cod tissues and during early ontogeny

**Figure 5:**
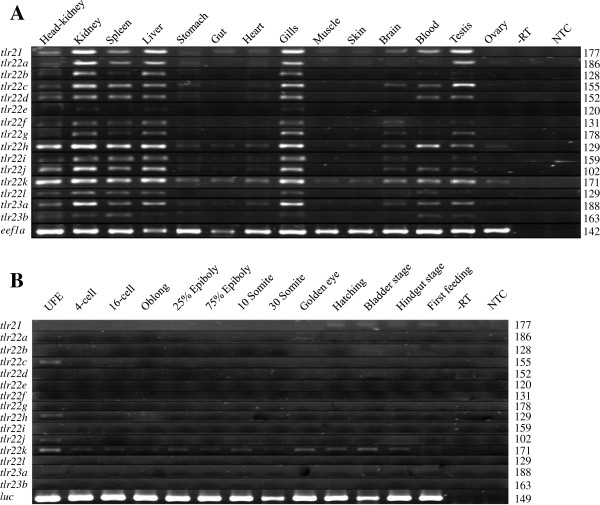
**Expression profile of cod teleost-specific *tlr*s in adult tissues and during early development. A**. Tissue specific expression of Atlantic cod *tlr21*, *tlr22* and *tlr23* genes. *Tlr*s are mainly expressed in immune-related tissues such as head-kidney, kidney, spleen, liver and gills. Transcripts of most paralogues were also found in high levels in blood and testis. *Eef1a* was used as an internal reference for RT-PCR. Minus reverse transcriptase (−RT) and no template (NTC) controls were included to ascertain the specificity of PCR primers. Amplicon sizes in bp are indicated on the right hand side of the figure. **B**. Expression analysis of *tlr*s during embryonic development. Low expression of *tlr21* was detected at later stages from hatching until first feeding, while *tlr23a* and *tlr23b* were not detected at any of the examined developmental stages. *Tlr22c*, *tlr22*, *tlr22j* and *tlr22k* transcripts were found in unfertilised eggs (UFE), while *tlr22k* was expressed at most developmental stages examined. *Luciferase* was used as an external reference for RT-PCR.

*Tlr21*, *tlr22* and *tlr23* paralogues were widely expressed across many tissues, including immune-related organs (head-kidney, kidney spleen and gills), liver and gonads (Figure 5A). All tissues examined, except ovary, had detectable levels of *tlr21* transcripts with high levels in kidney, liver, gills, testis and blood. A differential expression pattern across adult fish tissues was observed for *tlr22* paralogues. *Tlr22k* transcripts were detected in all tested tissues. *Tlr22e* had the lowest expression in kidney, liver and gills, while it was not detected in other tissues. All *tlr22* paralogues, except *tlr22e,* were detected in head-kidney, kidney, spleen, liver and gills at varied levels. Six out of 12 *tlr22* paralogues, *tlr22a*, *tlr22c*, *tlr22d*, *tlr22h*, *tlr22j* and *tlr22k*, were found to be expressed in stomach, while muscle and skin expressed only *tlr22k*. Testis had transcripts of most *tlr22* paralogues but *tlr22a*, *tlr22h* and *tlr22k* were the only genes to be detected in ovary. Within *tlr23* paralogues, expression of *tlr23b* was lower than that of *tlr23a* but they were both expressed in head-kidney, kidney, spleen, gills, blood and testis. *Tlr23a* transcripts were also found in liver, heart and brain.

*Tlr22c*, *tlr22h*, *tlr22j* and *tlr22k* transcripts were found in unfertilised eggs (Figure 5B). *Tlr22k* was the only *tlr22* paralogue to be expressed throughout early development and its transcripts were detected at epiboly, somite stage, golden eye, hatching, bladder and hindgut stages. Low expression of *tlr21* and *tlr22a* was detected at later stages from hatching until first feeding, while *tlr23a* and *tlr23b* were not present in any of the developmental stages examined.

### Differential expression following pathogen challenge

**Figure 6:**
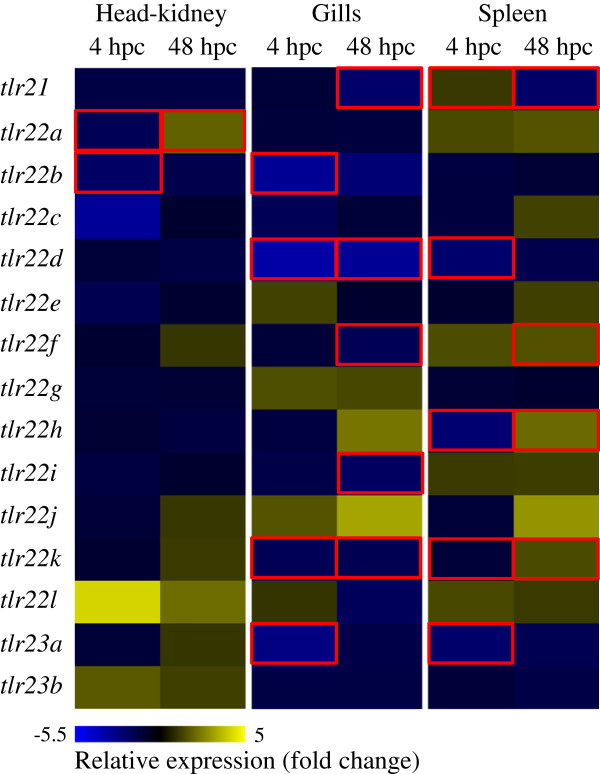
**Quantification of teleost-specific Atlantic cod *tlr*s in response to bath challenge with *V. anguillarum.*** Heatmap representing the expression of Atlantic cod *tlr*s in head-kidney, gills and spleen in response to bath challenge with *V. anguillarum*. After collecting initial control samples, fish were subjected to bath challenge with *V. anguillarum* strain H610 at a concentration of 2.6**·**10^7^ cfu·ml^-1^. Samples were collected at 4 (4 hpc) and 48 (48 hpc) h post-challenge. Relative expression of *tlr21*, *tlr22* and *tlr23* was determined by qPCR and expressed as ratios between each sample and the respective initial control. Significance levels were set at *P* < 0.05 and statistically different expression values are enclosed in red boxes. *Eef1a* and *ubi* were used as internal controls.

Teleost-specific *tlr*s in cod were differentially regulated following a bath challenge with *V. anguillarum* (Figure 6). A significant decrease of tlr21 expression was recorded after 48 h in gills (2.3-fold) and spleen (2.2-fold) compared to the initial control. In head-kidney, the highest change in expression was observed at 4 hpc in *tlr22c* (3.3-fold decrease) and *tlr22l* (4.2-fold increase), albeit not significant, while most of the other paralogues remained at basal levels. Following a 2-fold significant decrease in expression of *tlr22a* and *tlr22b* at 4 hpc in head-kidney, *tlr22a* transcripts reached a 2-fold higher expression at 48 hpc, which was also significant compared to the initial control levels. Several significant changes in expression of *tlr22* paralogues were also observed in gills and spleen following the bath challenge. In gills, *tlr22d* transcript levels were significantly reduced by 3.5-fold and this level was maintained through to 48 hpc. In the same tissue, a decrease of up to 2-fold in *tlr22k* expression was observed at 4 and 48 hpc. A significant decrease in *tlr22f* and *tlr22i* transcript levels was also observed at 48 hpc in gills. In spleen, *tlr22d* (2.4-fold), *tlr22h* (2.4-fold) and *tlr22k* (1.2-fold) were down-regulated at 4 hpc and an increase in expression of *tlr22f*, *tlr22h* and *tlr22k* (2.1-fold) was observed at 48 hpc compared to the initial control. Both *tlr23a* and *tlr23b* followed a similar pattern with significant reduction in the expression of *tlr23a* in gills (2.8-fold) and spleen (2.3-fold).

### Response to temperature stress

**Figure 7:**
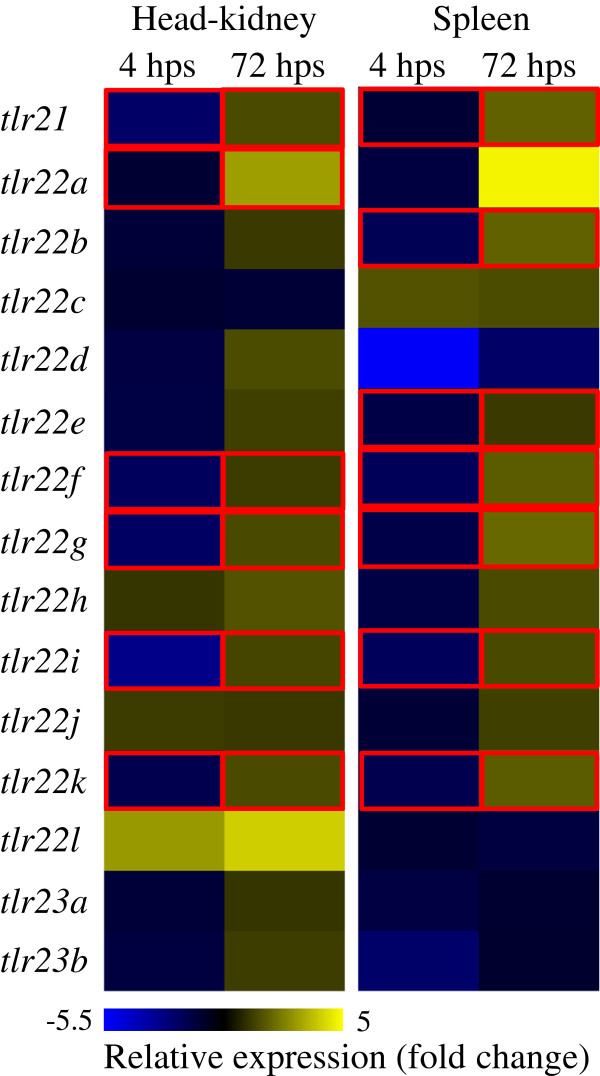
**Quantification of teleost-specific Atlantic cod *tlr*s in response to temperature stress.** Heatmap representing the expression of Atlantic cod *tlr*s in head-kidney and spleen in response to temperature stress. Adult fish were maintained at 4°C. After collecting initial control samples, the water temperature was gradually increased to 12°C in 4 h (4 hps) and the fish were maintained at this temperature for 72 h (72 hps). Relative expression of *tlr21*, *tlr22* and *tlr23* paralogues was quantified by qPCR as ratios between each sample and the initial control. Significance levels were set at *P* < 0.05 and statistically different expression values are enclosed in red boxes. *Eef1a* and *ubi* were used as internal controls.

Following thermal shock, a significant down-regulation of *tlr21* and *tlr22* paralogues was observed both in head-kidney and spleen, and most of the transcripts returned to initial levels or were up-regulated at 72 hps (Figure 7). In both organs, up to 3-fold significant reduction in *tlr21*, *tlr22f*, *tlr22g*, *tlr22i* and *tlr22k* mRNA levels was observed at 4 hps. *Tlr22a* transcript levels did not show much change to stress, but had a 3.1-fold increase at 72 hps in head-kidney. *Tlr22l* expression in head-kidney increased by 3-fold following thermal stress and then up to 4-fold at 72 hps, albeit not significant. The highest change in transcript levels was recorded for *tlr22d*, with a 5.5-fold decrease in spleen at 4 hps. No significant change was observed in *tlr23* expression with temperature stress.

### Molecular evolution of the cod tlr22 family

#### Tests of selection and relative rate tests

A pairwise codon based Z-test revealed that cod *tlr22* paralogues are evolving at different rates (Table 3). The highest dN-dS values were observed between *tlr22c* and *tlr22i* (2.852, *P* = 0.003) or *tlr22l* (2.787, *P* = 0.003). Even *tlr22c* and *tlr22d*, which are encoded by adjacent genes in the cod genome, were found to be evolving at different rates (dN-dS = 2.157, *P* = 0.016). Tajima’s relative rate test further confirmed the evolution of cod Tlr22 paralogues through pairwise comparison of these protein sequences with Tlr22b as outgroup. The test revealed that Tlr22d has undergone relatively high divergence compared to all other Tlr22 paralogues (Additional file 3).

**Table 3:** **Codon based Z-test of positive selection analysis between Atlantic cod *tlr22 *paralogues**

**Atlantic cod paralogues**	***tlr22b***	***tlr22c***	***tlr22d***	***tlr22f***	***tlr22g***	***tlr22h***	***tlr22i***	***tlr22j***	***tlr22l***
*tlr22b*		−0.436	−1.789	0.002	0.241	0.135	0.833	0.779	0.449
*tlr22c*	1.000		2.157	1.554	1.186	2.072	2.852	2.656	2.787
*tlr22d*	1.000	**0.016**		0.264	1.265	1.722	2.465	1.907	1.577
*tlr22f*	0.499	0.061	0.396		1.817	1.968	2.345	2.020	2.389
*tlr22g*	0.405	0.119	0.104	0.036		0.800	2.314	1.131	2.126
*tlr22h*	0.446	**0.020**	**0.044**	**0.026**	0.213		0.074	−0.427	0.306
*tlr22i*	0.203	**0.003**	**0.008**	**0.010**	**0.011**	0.471		1.632	1.893
*tlr22j*	0.219	**0.004**	**0.029**	**0.023**	0.130	1.000	0.053		0.901
*tlr22l*	0.327	**0.003**	0.059	**0.009**	**0.018**	0.380	**0.030**	0.185	

A modified Nei-Gojobori method with Jukes-Cantor correction was used. The test statistic (dN-dS) is shown above the diagonal and the corresponding P-value is indicated below the diagonal. P-values less than 0.05 are highlighted in bold. Positions containing gaps were eliminated for this analysis and in total 708 codons were included in the final dataset.

### Positive selection

**Figure 8:**
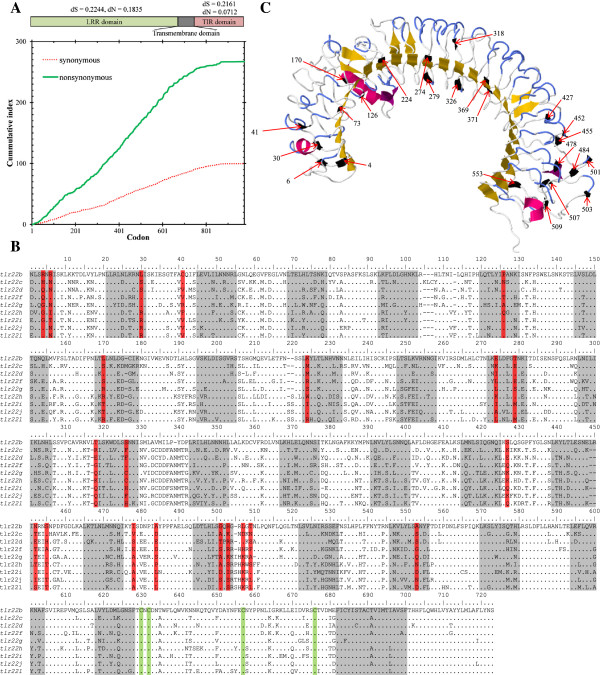
**Codons under positive selection in Atlantic cod Tlr22 paralogues and their location within Tlr22b. A**. Cumulative non-synonymous (green) and synonymous (red) substitutions for all pairwise comparisons between nine Atlantic cod *tlr22* paralogues. The ratio of non-synonymous (dN) over synonymous (dS) substitution is greater in the LRR region than in the TIR domain. **B**. Multiple sequence alignment of cod Tlr22. Amino acid residues identical to Atlantic cod Tlr22b are represented by a dot and alignment gaps are indicated by a dash. LRR regions are shaded in grey and positively selected sites are boxed in red. The cysteine cluster within the LRRCT domain is marked in green. **C**. Predicted structure of Atlantic cod Tlr22b. LRR region with the positively selected sites highlighted in black. Their amino acid position is indicated by arrows.

A sliding window analysis of the complete coding sequence of nine *tlr22* paralogues performed with SNAP revealed that the occurrence of non-synonymous mutations is not uniform throughout the coding sequence (Figure 8A). The average dN/dS ratio for the complete coding sequence was 0.748 (dS = 0.223, dN = 0.167), while the ratio for the LRR region was much higher (dN/dS = 0.815) than for the TIR region (dN/dS = 0.313). These differences in substitution rates confirm that the TIR domain within teleost-specific Tlrs in cod is more conserved than the LRR region. Thus, the site-specific positive selection analysis focused on the latter. Likelihood ratio tests (LRTs) revealed that PAML models that allowed for adaptive positive selection fitted the data better than those which did not (M3 versus M0, p = 0; M2 versus M1, p = 0; M8 versus M7, p = 0) (Table 4). In total, 24 positively selected codons (PSCs) were identified by all three models, M2, M3 and M8, with ω values of 4.08, 4.36 and 4.06, respectively. SLAC and FEL analyses found 2 and 28 codons evolving under positive selection with p-value less than 0.1 (data not shown) and REL identified 19 sites PSCs with Bayes factor greater than 50 (Table 4). In total, the Datamonkey server analysis indicated 37 codons to be under selection pressure. The 24 sites indicated by the Bayesian approach using PAML were also selected by Datamonkey. All codons under positive selection were found within the N-terminal LRR domain, which recognises pathogens and 19 of these sites were present on the convex surface (Figure 8B, 8C). Fifteen of the 24 PSCs were found within the LRR repeats. Only five of the 24 sites were found in beta sheets within the concave surface of the horseshoe-shaped domain, while most of the amino acids under selection pressure were on the structural components of the LRRs, the coils.

**Table 4:** **Identification of positively selected sites in Atlantic cod *tlr22 *paralogues by maximum likelihood analysis**

**Models**	**Parameter estimates**	**Ln likelihood**	**Model comparison**	**Positively Selected sites**
M0: neutral	ω = 1.12	−10057.27		None
M1: nearly neutral	ω_0_ = 0.081, ω_1_ = 1	−9946.92		Not allowed
	p_0_ = 0.39, p_1_ = 0.61			
M2: positive selection	ω_0_ = 0.05, p_0_ = 0.28	−9850.74	M2 vs M1	4, 6, 30, **41**, **73**, 126, **170**, **224**, 250, **274**, **279**, **318**, **326**, **333**, **369**, 371, 427, 443, 452, **455**, 458, 478, **484**, 501, **503**, 505, 507, **509**, 528, 531, **553**, 577, 674
	ω_1_ = 1, p_1_ = 0.54		2ΔlnL = 192.35,	
	**ω**_**2**_**= 4.08**, p_2_ = 0.18		df = 2, p = 0	
M3: discrete	ω_0_ = 0.18, p_0_ = 0.35	−9850.56	M3 vs M0	**1**, **3**, **4**, **6**, 9, 11, 12, **13**, **16**, **18**, 23, 25, **27**, **28**, **30**, 33, **37**, **40**, **41**, **43**, 44, **49**, **51**, 52, **54**, **56**, **57**, **59**, 68, 70, 71, **73**, **75**, **76**, **78**, **80**, 81, 82, 83, **94**, 95, 97, **108**, **110**, 112, **115**, 116, **122**, **124**, **126**, 127, **129**, 132, 134, **147**, 152, 154, **156**, **157**, 159, 163, 169, **170**, **172**, **174**, 175, 177, **178**, **179**, 180, 181, 189, 194, **196**, **197**, **210**, 211, 212, 215, **224**, 227, **230**, **231**, 233, **234**, **236**, **237**, **240**, **241**, **245**, **246**, **247**, **250**, 251, **252**, 253, 257, **260**, 262, **267**, **268**, 271, **273**, **274**, 276, 277, **279**, 281, **284**, **287**, 288, 294, **295**, **297**, 298, 301, **302**, **303**, **305**, 307, **311**, **313**, **315**, 316, **318**, **320**, **326**, 330, **331**, **333**, 334, 335, **336**, **338**, 339, **340**, 341, **342**, **344**, **345**, **347**, **349**, **350**, **352**, **354**, **355**, **357**, 358, 368, **369**, **371**, 375, **376**, **378**, **379**, 382, 383, 387, 388, **392**, **393**, **395**, **397**, **400**, **402**, **403**, **406**, **410**, **413**, 414, **416**, **417**, 419, 421, **426**, **427**, 428, **429**, **430**, 432, **434**, **438**, 440, 441, **443**, 445, **448**, **452**, 453, **455**, **457**, **458**, **460**, **471**, **472**, **474**, 475, **476**, **478**, 480, **481**, **484**, **493**, **495**, **498**, 499, **501**, **503**, **504**, **505**, **506**, **507**, **508**, **509**, **510**, **517**, **524**, 526, **528**, **529**, **530**, **531**, **532**, **533**, **535**, **538**, **539**, **548**, 549, **550**, 551, 552, **553**, **555**, **556**, 557, 572, **574**, **577**, **578**, **579**, 595, **596**, 598, **600**, **601**, **603**, **613**, **616**, **619**, **620**, **622**, **624**, **639**, **642**, **643**, **645**, **650**, 662, 666, 670, **673**, **674**, **680**, **681**, **691**, 702, **712**, 719
	ω_1_ = 1.19 , p_1_ = 0.49		2ΔlnL = 413.43,	
	**ω**_**2**_**= 4.36**, p_2_ = 0.16		df = 4, p = 0	
M7: β	p = 0.02, q = 0.01	−9951.60		Not allowed
M8: β + ωS>1	p = 0.1, q = 0.05	−9850.86	M8 vs M7	1, 4, **6**, 30, **41**, 56, **73**, **126**, **170**, 174, **224**, 245, 250, **274**, **279**, 287, 295, **318**, **326**, **333**, 342, 344, 355, **369**, **371**, 378, 393, 400, 427, **443**, **452**, **455**, 457, 458, 460, 471, 474, **478**, **484**, **501**, **503**, 505, 506, **507**, **509**, **528**, 529, 530, **531**, **553**, **577**, 619, **674**
	**ω = 4.06**		2ΔlnL = 201.48,	
	p_0_ = 0.81, p_1_ = 0.19		df = 2, p = 0	
REL				4, **6**, 30, **41**, 49, **73**, 76, 126, 147, 157, **170**, **224**, 246, **274**, **279**, 295, **318**, 320, **326**, **333**, 342, **369**, **371**, 397, 400, **427**, **452**, 455, 478, **484**, 501, **503**, **507**, **509**, **553**, 578, 613

Only positively selected sites with Bayesian posterior probabilities above 95% are indicated and the ones greater than 99% are highlighted in bold.

In the REL analysis, positively selected sites with a Bayes factor greater than 50 are highlighted in bold.

## Discussion

We have characterised the full-repertoire of the highly expanded teleost-specific *tlr* family in Atlantic cod, which includes one *tlr21*, twelve *tlr22* and two *tlr23* genes encoded by its genome. Phylogenetic analysis of *tlr* paralogues from 15 teleost species recovered monophyly of all *tlr22* paralogues, suggesting their origin from a common teleost ancestor. All cod *tlr22* paralogues were grouped under a single clade, which indicates that they have likely arisen through tandem duplications. Cod is the first sequenced vertebrate identified to have lost all the mammalian cell surface and bacterial recognising TLR orthologues [14]. Based on the knowledge of the functional coverage of the vertebrate TLRs, 10 TLRs are predicted to be present in the common vertebrate ancestor, namely, TLR2, 3, 4, 5, 7, 8, 9, 11, 21 and 22 [37]. Genes encoding Tlr2, Tlr4, Tlr5 and Tlr11 are absent, while Tlr3, Tlr7-9 are intracellular Tlrs. Hence, Tlr21 and Tlr22 are the only plausible cell surface Tlrs encoded by the cod genome.

Partial synteny analysis based on the current genome build revealed conservation between Tlr22 encoding genes in cod and those in stickleback, tiger pufferfish and green-spotted pufferfish, within the genomic region containing *sh3kbp1* and *map3k15* genes. Sh3kbp1 (SH3-domain kinase binding protein 1) is an adapter protein involved in regulating diverse signal transduction pathways, while Map3k15 (mitogen-activated protein kinase kinase kinase 15) plays a key role in signal transduction and is essential for stress-induced apoptosis [38]. Several *tlr22* paralogues are in close proximity within the cod genome and seem to have arisen through tandem duplications. There is no uniform exon-intron structure within these *tlr22* paralogues. Full length CDS of cod *tlr22b*, *tlr22d*, *tlr22g* and *tlr22i* are encoded by 5, 3, 3 and 3 exons, respectively. In the case of goldfish (*Carassius auratus*), zebrafish and rainbow trout (22 and 22 l) *tlr22* has a single exon, while the tiger pufferfish and large yellow croaker orthologues are encoded by four, three and three exons, respectively [10,39,40]. *Tlr22* genes in basal teleosts such as Cyprinidae and Salmonidae are represented by a single exon, while their orthologues in higher teleosts (Sciaenidae, Tetraodontidae and Gadidae) contained multiple exons. This suggests that Tlr22 may have been encoded by an uninterrupted exon in the common vertebrate ancestor and has acquired additional introns during the evolution. According to homology, synteny and phylogenetic analyses, *tlr22a* encoded by a single exon (based on partial sequence) seems to be the ancestral Tlr22 encoding gene and the remaining eleven paralogues have arisen through tandem duplications. It was not possible to perform a synteny analysis for *tlr21* and *tlr23* paralogues, since the corresponding genomic scaffolds were short and did not contain more than two genes. Cod *tlr21* is represented by an uninterrupted exon in the genome, sharing this gene structure with zebrafish, tiger pufferfish and stickleback (*tlr21a*) homologues, while stickleback (*tlr21b*), channel catfish (*Ictalurus punctatus*) and medaka homologues are encoded by multiple exons. *Tlr23* has been identified in two more teleosts, tiger pufferfish and green-spotted pufferfish, both comprising three exons each, while cod *tlr23a* had five exons and *tlr23b* is composed of three exons in its genome. Completion of current genome build will provide a better understanding of the origin of the various paralogues as well as synteny with other teleosts.

TLRs have cysteine clusters flanking either side of the LRR region with two to five cysteine residues, which are denoted LRRCT and LRRNT domains [25]. While LRRNT regions are variable among TLRs, LRRCT contains a highly conserved consensus sequence and is known to play a crucial role in TLR signalling. The LRRCT forms a compact structure stabilised by disulphide bridges positioning the extracellular domain of the TLR relative to the membrane, as seen in the structure of human TLR3 protein [41]. Similar to other known teleost Tlr21s, the Atlantic cod Tlr21 protein has a CxCx_24_Cx_15_C motif at its LRRCT domain, while Tlr22 and Tlr23 had a CxCx_24_Cx_18_C motif in their LRRCT, characteristic of teleost Tlr22 proteins [11,25]. The vertebrate TLR N-terminal ectodomain is made up of several LRRs and is involved in recognising PAMPs. The ectodomain of the teleost-specific Tlrs in cod is made of up to 27 LRR repeats [25]. Full length CDS of two *tlr23* paralogues encoded for proteins containing 27 (Tlr23a) and 14 (Tlr23b) LRRs within their N-terminal domain. It is noteworthy that cod Tlr23b contains such a low number of LRRs, since vertebrate Tlrs contain 16 to 28 LRRs. As cod is the first vertebrate known to encode for two *tlr23* paralogues, it is likely that *tlr23a* is the ancestral gene and Tlr23b has lost LRRs during evolution. Homology modelling of Tlr22b based on human TLR3 ectodomain (PDB ID: 2A0Z) [41] revealed a characteristic horseshoe-shaped structure. The human TLR3 ectodomain is composed of 23 LRRs forming the classical horseshoe-shaped structure and the concave inner surface is composed of 21 parallel beta sheets with the hydrophobic residues pointing inwards forming a hydrophobic core. In the cod Tlr22b model, LRR22 formed an external protrusion similar to human LRR20. LRR11 formed a very large regular alpha helix and protruded outwards similar to human LRR12. These two LRRs may be involved in the recognition of PAMPs as observed for LRR12 and LRR20 in human TLR3.

A similar pattern of cod *tlr21*, *tlr22* and *tlr23* expression was observed in zebrafish *tlr21* and *tlr22*[11], channel catfish *tlr21*[42], rainbow trout *tlr22* and *tlr22l*[12], large yellow croaker (*Larimichthys crocea*) *tlr22*[39], grass carp (*Ctenopharyngodon idella*) *tlr22*[40], goldfish *tlr22*[13] and orange-spotted grouper *tlr21*[43]. Nevertheless, the differential expression pattern observed across tissues for *tlr22* paralogues indicates that this gene may have diversified to attain specific roles in different tissues of cod. To date, cod and grass carp [40] are the only two teleosts known to express a *tlr22* paralogue in fast muscle. Cod *tlr22k* was expressed in skin, similarly to channel catfish *tlr22*[42]. The skin is an important mucosal defence organ [28] and the presence of *tlr22k* transcripts may trigger the innate immune response by detecting PAMPs, once they cross the mucosal layer into the skin. Cod testis expressed most *tlr*s, similarly to zebrafish *tlr22*[11]. Several mammalian TLRs in mouse are reported to be involved in the testicular innate immune response especially in Sertoli cells [44]. Thus, *tlr22* expression in testis suggests that it may be involved in protecting the male reproductive tract in cod and other teleosts. Teleost-specific *tlr*s showed varied developmental expression patterns and unfertilised eggs had *tlr22c*, *tlr22h* and *tlr22k* transcripts, possibly derived from maternal source. Historically, *Drosophila toll* was identified as a key player in specification of the dorso-ventral axis during embryonic development and several *toll* genes were found to be expressed throughout the developmental stages [45]. The main focus of mammalian TLR research is on the immune function of the gene and less evidence of their role in embryogenesis is established in vertebrates. A recent study on mouse brain has identified specific expression patterns of TLR7 and TLR9 expression in developing brain, which has been linked to the development of the central nervous system of vertebrates [46]. In grass carp, *tlr22* transcripts were also found during late developmental stages [47]. This study corroborates our data, suggesting that teleost-specific *tlr*s may also play a role in embryogenesis.

Tiger pufferfish Tlr22 was originally thought to be a functional substitute of human TLR3, as it responds to dsRNA and may therefore promote antiviral protection in teleosts [10]. Several *in vivo* and *in vitro* studies have shown that teleost-specific *tlr*s do respond to a wide variety of PAMPs originating from bacteria and parasites [4]. An increase in expression of *tlr22* was found in LPS stimulated macrophages as well as in LPS, *Aeromonas salmonicida* or *Mycobacterium cheloni* stimulated leucocytes in goldfish [12]. LPS, peptidoglycan and poly(I:C) as well as *M. marinum* up-regulated expression of *tlr22* in larvae and adult zebrafish, respectively [11,48]. Continuous exposure of rainbow trout PBL, spleen and kidney to inactivated *A. salmonicida*, induced up to 8-fold increase in expression of *tlr22* and *tlr22l* after 24 h. Following stimulation with poly(I:C), high levels of *tlr22* transcripts in spleen of large yellow croaker [39]. A similar effect was seen on the expression of *tlr22* in grass carp infected with reovirus [40]. In the present study, bath challenge with *V. anguillarum* induced a 2.1-fold increase of *tlr22f*, *tlr22h* and *tlr22k* transcript levels in spleen at 48 hpc compared to the initial control. In general, most of the genes analysed in this study responded to bacterial bath challenge across the three tissues that were examined, but with some tissue-specific responses. In particular, *tlr22f* and *tlr22k* were down-regulated in gills but up-regulated in spleen at 48 hpc. Our data revealed that in addition to recognising dsRNA, teleost-specific *tlr*s respond to PAMPs from bacterial origin.

*Tlr22d* was down-regulated by 5.5-fold in spleen following exposure to high temperature, indicating that it may be involved directly in the immune response to heat shock. Heat stress is known to induce an innate immune response by activating the overexpression of various heat shock proteins. In mammals, TLR2 and TLR4 up-regulation is mediated by p38-kinase and might be involved in the enhanced response of PAMP in humans monocytes induced by head shock [49]. In the thermal shock experiment, heat shock protein 70 (*hsp70*) was up-regulated in skin by 3-fold at 72 hps (data not shown), thus confirming the effect of the temperature stress. Human HSP70 released into the extracellular milieu binds to TLR2 and TLR4 and exerts immunoregulatory effects through its chaperokine activity [50]. HSP60 is also found to be dependent on TLR4 for induction of specific cytokines [51]. Most cod teleost-specific *tlr* genes were differentially regulated in this temperature stress experiment, indicating that they may be involved in regulating the heat shock as well as the immune response.

In our previous study, we demonstrated that *tlr22* genes from several teleost taxa (Cyprinidae, Gasterosteidae, Salmonidae, Adrianichthyidae, Tetraodontidae) are under adaptive selection pressure [11]. Pairwise comparison of N-terminal LRR domains amongst cod *tlr22* paralogues showed that they are evolving at different rates. Significant dN-dS values greater than one were observed for most comparisons, the highest being between *tlr22c* and *tlr22i*. Similar results were obtained from Tajima’s relative rate test, which in fact revealed that *tlr22d* is evolving considerably faster than all other paralogues. This accelerated divergence may account for its involvement in the heat shock response, since *tlr22d* was significantly down-regulated following thermal stress.

Average non-synonymous nucleotide substitutions within Atlantic cod *tlr22* paralogues were generally much higher than synonymous ones, especially within the LRR coding region. Five PSCs at positions 73, 170, 274, 369 and 371 were found within the β sheets forming the hydrophobic core of the TLR ectodomain [52]. At positions 73, 170 and 369, all nine cod Tlr22 paralogues predominantly contained a hydrophilic residue, while 274 and 371 were mostly hydrophobic in nature. PSCs 427, 452, 455 and 509 are present after LRR19, LRR20, LRR20 and LRR22, respectively, and are generally represented by different hydrophilic amino acids, where hydrophobic residues are normally found [53]. LRR11 and LRR22, which protrude outwards from the horseshoe-shaped domain that recognises PAMPs, have one and four PSCs, respectively. Ligand specificity may be based on variations in the amino acids in the solvent-exposed beta sheets or on variations in the convex surface of the horseshoe-shaped domain [53]. The substitution of hydrophilic amino acids for hydrophobic ones in and around the beta sheets will affect the polarity of the core. Also, changes i in other PSCs may be altering the polarity and the structure of the ectodomain, thus producing striking variations in the PAMP recognising sites of Tlr22 paralogues in cod. Four PSCs were found to be unique to Tlr22d, which seems to be involved in the heat shock response. Unlike other Tlr22 isoforms, the first three sites of Tlr22d had a negative charge (E318, E427 and E452) while the fourth was positive (H455).

Positive selection within duplicate genes has been related to their functional diversification through neofunctionalisation [54,55]. Our study revealed that several PSCs in cod *tlr22* genes may produce striking changes in critical protein sites and may therefore be associated with adaptation to evolving pathogens or acquisition of additional functions, such as the heat shock response. Hence, it is likely that these duplicate *tlr22* genes are undergoing neofunctionalisation. Taken together with the observed asymmetric evolution rates amongst cod *tlr22* paralogues, our data favour the adaptation model, as opposed to the Dykhuizen-Hartl model (reviewed in [19]).

## Conclusion

We have identified and annotated 15 *tlr* genes representing all the members of the highly expanded teleost-specific Tlr family in Atlantic cod, which includes 12 *tlr22* paralogues. They seem to have evolved through lineage-specific tandem duplications, perhaps to compensate for the absence of bacterial recognising and other cell surface Tlrs. The various *tlr22* paralogues are evolving at different molecular rates and several codons in the region coding for their ligand binding domain are under adaptive selection, which may contribute to their functional diversification through neofunctionalisation. This conclusion is corroborated by experimental evidence of differential expression upon thermal shock and bacterial challenge.

## Competing interests

The authors declare no competing interests, financial or otherwise, regarding this manuscript.

## Authors’ contributions

AYMS, JMOF and VK designed the experiment and wrote the manuscript. Experiments were performed by AYMS, who analysed the data jointly with JMOF. JMOF, VK and JD reviewed the paper draft. All authors approved the final version of the manuscript.

## Supplementary Material

Additional file 1 **Primers used for sequencing of teleost-specific*tlr*s in Atlantic cod.** Click here for file

Additional file 2 **List of teleost-specific *tlr*s used for phylogenetic analysis.** Click here for file

Additional file 3 Tajima’s relative test for the comparison of evolutionary distance between Atlantic cod Tlr22 paralogues. Click here for file
