# Supplementary material for: Diversification of the expanded teleost-specific toll-like receptor family in Atlantic cod, Gadus morhua
Source: BMC Evol Biol. 2012 Dec 29;12:256. doi: 10.1186/1471-2148-12-256 (PMC3549756; doi:10.1186/1471-2148-12-256)
Supplement: Additional file 1 — Primers used for sequencing of teleost-specifictlrs in Atlantic cod. [file 1471-2148-12-256-S1.pdf]

Supplementary table 1. Primers used for sequencing of teleost-specific *tlr*s in Atlantic cod

Gene name	Accession number	Sequencing primers (Forward and Reverse) (5'-3')	5' RACE primer (Outer and Nested) (5'-3')	3' RACE primer (Outer and Nested) (5'-3')
tlr21	JX074771	GGGATACATTACTAATCTCATACACTGC<br>GCAGAGTCGGTTTGGTCTCCT	-	-
tlr22a	JX074772	ACATTCCAGCAAGGGTGACGA<br>AGGAACCACGCCTTGTCAC	-	-
tlr22b	JX074773	GGTTCGCTCCACATCACA<br>CGTCCTTCTGCTCGTCAA	TTCGTCCCAAAGTCCAACTCTTTCAGATT<br>CGGACTTTAAGAGATGTCAGTGATGGGATT	TTCCAGCCAGGCAGACCTATAATAGAAAAC<br>CTGTGTGGTCAGTCGTGATTACCTGC
tlr22c	JX074774	TCACACGGAAGACGGAGGC<br>CAGAGGGTGTTCGGGATAGG	-	-
tlr22d	JX074775	AAACGGAAATGGCGATGC<br>CTTTGAGGTCTGGAGGGTAGTT	CATTGAAACTATTCTTCTGACAGATGGC<br>CAATCCTTCAGCATGTAACCAGCAGC	CCTGAGGTCTCTCGATTTTCTGCTCCTT<br>ACAACAATCAAACACAAGTGTATGATGCGT
tlr22e	JX074776	GGAGGGTATGTTTGATGGCTTAG<br>CTTGGAGATTTGGCAGGTGTT	GGACAGGTATTGTAAATGTGGTGTATG<br>TGTTTTGATCCTATTGGAAGTCAGATGC	-
tlr22f	JX074777	CGCTTAGACCTGAGACACAACTT<br>CGGTGGTGCAGACACAGTCTC	AGTTTGCTCAGAGACTTGAAAGAGG<br>GATCAAGTTGTGTCTCAGGTCTAAGCG	-
tlr22g	JX074778	AGAGCGTATTTCTTATCCACCATT<br>GAAGGCGTCGTATTGATGAGC	CTTGCTCAGAGACTTGAAAGAGGC<br>GGCTGGTGCTACTGTTTGGATCTGAT	AATGGGTAATCAGCAACAAGCAAACACAA<br>GGCATCTGGTCTATGCGTACTACCTCATG
tlr22h	JX074779	CATTCCAGAAAGAGTCAGAAGTA<br>TTCACTCTCCAGGTAATCGC	TCAAACATACCCTCCTGTAGCTTACAAAGG<br>AACACAAAAGTCGCAGATTCAATCTTGGAG	-
tlr22i	JX074780	AGAGTCACAAGTATAGAACTG<br>TCTCCCAGAACAGGTTTG	TCTTGGAGATCCTGTTGCTTGTCAG<br>TGGTGTTTCCAGACAGTTCTATACTTG	AGCCAGGTAAACCGATCATGGAAAACATTA<br>TGCAGCAACTGTCTCCGTACTACCG
tlr22j	JX074781	CCCGTCTCCAAGGTCTCTATC<br>GAAGGCGTCGTATTGATGAGC	GGTGTGTTTTCTTTGAAGGCTCCGTT<br>GGACGAGAGAGGATTATTGTATAGATAGA	-
tlr22k	JX074782	TCCTACAATGGCAACTGGTCTAC<br>CCCAGCCCTCGTCGTTTG	-	GGACTTCATCTGTTATATTTCAACGGCA<br>TGACCCTAGCAGTCTCCTTCACCC
tlr22l	JX074783	ATGCCATGTAGGGGAATTCGT<br>ATGCCGTTGAAATATAACAGATGAA	-	CTTGGGGATGAATGAGATAACTGAA<br>ATTCGTTTCAATCTCTTAGGCTGCTTA
tlr23a	JX074784	TCTCCAATCGCATCTCCAATC<br>ACTCGCTCTCCAGGTAGTGTCT	GAAATCTAAGCAGCCTACCTCAGCAAT<br>GAGAGAGTTACCTGCCAAATCCAAGT	AGGACGAGCGAGGCTGGAGACT<br>GCACCACCGAGACTTCCAGCCAGG
tlr23b	JX074785	GACCTGTGACTGCTCCAACG<br>GCGGTGCTGCTCATTATTCTT	GCCCACAGCAGGAAGCCAGCGT<br>CAGCGTTGGAGCAGTCACAGGTCA	ACTGTTCACTTTACTCGCTTCCTTCA<br>GAGAGGAAGAATAATGAGCAGCACC
