# Supplementary material for: Diversification of the expanded teleost-specific toll-like receptor family in Atlantic cod, Gadus morhua
Source: BMC Evol Biol. 2012 Dec 29;12:256. doi: 10.1186/1471-2148-12-256 (PMC3549756; doi:10.1186/1471-2148-12-256)
Supplement: Additional file 2 — List of teleost-specific tlrs used for phylogenetic analysis. [file 1471-2148-12-256-S2.pdf]

Supplementary table 2. List of teleost-specific *tlr*s used for phylogenetic analysis

Gene Name	Accession number (GenBank/Ensembl)	Organism	Family	Length (bp)
tlr21	ENSORLT00000016853	*Oryzias latipes*	*Adrianichthyidae*	2690
tlr21	NM_001199335	*Danio rerio*	*Cyprinidae*	2970
tlr21	JX074771	*Gadus morhua*	*Gadidae*	3047
tlr21a	ENSGACT00000012366	*Gasterosteus aculeatus*	*Gasterosteidae*	2784
tlr21b	ENSGACT00000011120	*Gasterosteus aculeatus*	*Gasterosteidae*	2748
tlr21	DQ529277	*Ictalurus punctatus*	*Ictaluridae*	2961
tlr21a	JF738115	*Epinephelus coicodes*	*Serranidae*	2940
tlr21b	JF738114	*Epinephelus coicodes*	*Serranidae*	2928
tlr21	NM_001032579	*Takifugu rubripes*	*Tetraodontidae*	2898
tlr22	ENSORLT00000025295	*Oryzias latipes*	*Adrianichthyidae*	2917
tlr22	AY162178	*Carassius auratus*	*Cyprinidae*	2838
tlr22	HQ452813	*Cyprinus carpio*	*Cyprinidae*	2838
tlr22	HQ676542	*Ctenopharyngodon idella*	*Cyprinidae*	2865
tlr22	BC163527	*Danio rerio*	*Cyprinidae*	2844
tlr22a	JX074772	*Gadus morhua*	*Gadidae*	1654
tlr22b	JX074773	*Gadus morhua*	*Gadidae*	3406
tlr22c	JX074774	*Gadus morhua*	*Gadidae*	2408
tlr22d	JX074775	*Gadus morhua*	*Gadidae*	3252
tlr22e	JX074776	*Gadus morhua*	*Gadidae*	1612
tlr22f	JX074777	*Gadus morhua*	*Gadidae*	2707
tlr22g	JX074778	*Gadus morhua*	*Gadidae*	3082
tlr22h	JX074779	*Gadus morhua*	*Gadidae*	2847
tlr22i	JX074780	*Gadus morhua*	*Gadidae*	3219
tlr22j	JX074781	*Gadus morhua*	*Gadidae*	2149
tlr22k	JX074782	*Gadus morhua*	*Gadidae*	384
tlr22l	JX074783	*Gadus morhua*	*Gadidae*	2706
tlr22	ENSGACT00000007214	*Gasterosteus aculeatus*	*Gasterosteidae*	2878
tlr22	HQ677725	*Ictalurus punctatus*	*Ictaluridae*	2862
tlr22	JN969981	*Siniperca chuasti*	*Percichthyidae*	2871
tlr22	AJ628348	*Oncorhynchus mykiss*	*Salmonidae*	2922
tlr22l	AJ878915	*Oncorhynchus mykiss*	*Salmonidae*	2910
tlr22a1	AM233509	*Salmo salar*	*Salmonidae*	2916
tlr22a2	FM206383	*Salmo salar*	*Salmonidae*	2778
tlr22b	BT045774	*Salmo salar*	*Salmonidae*	2888
tlr22	GU324977	*Larimichthys crocea*	*Sciaenidae*	2898
tlr22	AB197916	*Takifugu rubripes*	*Tetraodontidae*	2808
tlr22	ENSTNIT00000016840	*Tetraodon nigroviridis*	*Tetraodontidae*	2854
tlr23a	JX074784	*Gadus morhua*	*Gadidae*	3427
tlr23b	JX074785	*Gadus morhua*	*Gadidae*	2165
tlr23	AC156435	*Takifugu rubripes*	*Tetraodontidae*	2826
tlr23	ENSTNIT00000008231	*Tetraodon nigroviridis*	*Tetraodontidae*	2821
