# Supplementary material for: Diversification of the expanded teleost-specific toll-like receptor family in Atlantic cod, Gadus morhua
Source: BMC Evol Biol. 2012 Dec 29;12:256. doi: 10.1186/1471-2148-12-256 (PMC3549756; doi:10.1186/1471-2148-12-256)
Supplement: Additional file 3 — Tajima’s relative test for the comparison of evolutionary distance between Atlantic cod Tlr22 paralogues. [file 1471-2148-12-256-S3.pdf]

Supplementary table 3. Tajima's relative test for the comparison of evolutionary distance between Atlantic cod Tlr22 paralogues

A	B	Identical sites in all three sequences	Divergent sites in all three sequences	Unique differences in Sequence A	Unique differences in Sequence B	Unique differences in Sequence C (Tlr22b)	Chi-square values	P-value
Tlr22c	Tlr22d	450	51	77	53	79	**4.43**	**0.035**
	Tlr22f	435	69	68	69	70	0.01	0.932
	Tlr22g	429	73	68	74	66	0.25	0.615
	Tlr22h	421	78	64	83	65	2.46	0.117
	Tlr22i	421	82	68	83	56	1.49	0.222
	Tlr22j	429	73	65	75	69	0.71	0.398
	Tlr22l	420	76	72	84	59	0.92	0.337
Tlr22d	Tlr22f	453	56	49	74	78	**5.08**	**0.024**
	Tlr22g	441	69	55	86	58	**6.82**	**0.009**
	Tlr22h	431	69	53	96	61	**12.41**	**0.0004**
	Tlr22i	427	68	61	100	53	**9.45**	**0.002**
	Tlr22j	435	60	58	92	65	**7.71**	**0.005**
	Tlr22l	430	57	61	97	65	**8.20**	**0.004**
Tlr22f	Tlr22g	439	65	58	63	85	0.21	0.649
	Tlr22h	430	61	55	73	92	2.53	0.111
	Tlr22i	435	74	54	68	79	1.61	0.205
	Tlr22j	438	60	56	65	92	0.67	0.413
	Tlr22l	429	58	63	74	87	0.88	0.348
Tlr22g	Tlr22h	432	67	53	65	93	1.22	0.270
	Tlr22i	432	77	56	65	79	0.67	0.413
	Tlr22j	439	70	54	58	89	0.14	0.705
	Tlr22l	430	71	61	67	81	0.28	0.596
Tlr22h	Tlr22i	449	56	40	36	129	0.21	0.646
	Tlr22j	457	47	37	28	142	1.25	0.264
	Tlr22l	449	57	43	36	126	0.62	0.431
Tlr22i	Tlr22j	452	58	42	37	121	0.32	0.574
	Tlr22l	446	55	46	43	120	0.10	0.750
Tlr22j	Tlr22l	458	42	34	36	141	0.06	0.811

Chi-square values are provided for pairwise comparison with the *P*-values enclosed within brackets. *P*-value less than 0.05 is used to reject the null hypothesis of equal rates between lineages and are shown in bold.
